# TH-302-loaded nanodrug reshapes the hypoxic tumour microenvironment and enhances PD-1 blockade efficacy in gastric cancer

**DOI:** 10.1186/s12951-023-02203-8

**Published:** 2023-11-22

**Authors:** Zhixiong Wang, Menglin Zhu, Runyu Dong, Danping Cao, Yanna Li, Zhiqiang Chen, Juan Cai, Xueliang Zuo

**Affiliations:** 1 Department of Gastrointestinal Surgery, The First Affiliated Hospital, Yijishan Hospital of Wannan Medical College, Wuhu, 241001 China; 2 Hepatobiliary Center, The First Affiliated Hospital of Nanjing Medical University, Key Laboratory of Liver Transplantation, Chinese Academy of Medical Sciences, NHC Key Laboratory of Living Donor Liver Transplantation, Nanjing, China; 3 Anhui Province Key Laboratory of Non-coding RNA Basic and Clinical Transformation, Wannan Medical College, Wuhu, 241001 China; 4 Department of Oncology, The First Affiliated Hospital, Yijishan Hospital of Wannan Medical College, Wuhu, 241001 China

**Keywords:** TH-302, Nanodrug, Hypoxia, Immune escape, PD-1 blockade

## Abstract

**Background:**

Hypoxia, a common characteristic of the tumour microenvironment, is involved in tumour progression and immune evasion. Targeting the hypoxic microenvironment has been implicated as a promising antitumour therapeutic strategy. TH-302 can be selectively activated under hypoxic conditions. However, the effectiveness of TH-302 in gastric cancer combined immunotherapy remains unclear.

**Methods:**

We designed mPEG-PLGA-encapsulated TH-302 (TH-302 NPs) to target the hypoxic area of tumour tissues. A particle size analyzer was used to measure the average size and zeta potential of TH-302 NPs. The morphology was observed by transmission electron microscopy and scanning electron microscopy. The hypoxic area of tumour tissues was examined by immunofluorescence assays using pimonidazole. Flow cytometry analysis was performed to measure the levels of TNF-α, IFN-γ, and granzyme B. The synergistic antitumour activity of the combination of TH-302 NPs with anti-PD-1 (α-PD-1) therapy was assessed in vitro and in vivo. Haematoxylin and eosin staining of major organs and biochemical indicator detection were performed to investigate the biological safety of TH-302 NPs in vivo.

**Results:**

TH-302 NPs inhibited the proliferation and promoted the apoptosis of gastric cancer cells under hypoxic conditions. In vitro and in vivo experiments confirmed that TH-302 NPs could effectively alleviate tumour hypoxia. TH-302 NPs exhibited high bioavailability, effective tumour-targeting ability and satisfactory biosafety. Moreover, the combination of TH-302 NPs with α-PD-1 significantly improved immunotherapeutic efficacy in vivo. Mechanistically, TH-302 NPs reduced the expression of HIF-1α and PD-L1, facilitated the infiltration of CD8^+^ T cells and increased the levels of TNF-α, IFN-γ, and granzyme B in tumours, thereby enhancing the efficacy of α-PD-1 therapy.

**Conclusion:**

TH-302 NPs alleviated the hypoxic tumour microenvironment and enhanced the efficacy of PD-1 blockade. Our results provide evidence that TH-302 NPs can be used as a safe and effective nanodrug for combined immunotherapy in gastric cancer treatment.

**Supplementary Information:**

The online version contains supplementary material available at 10.1186/s12951-023-02203-8.

## Introduction

Gastric cancer ranks fifth in cancer incidence and fourth in cancer mortality worldwide, posing a great threat to human health [1]. Early gastric cancer is usually asymptomatic, and most patients are diagnosed at an advanced stage. The 5-year survival rate of advanced gastric cancer patients remains poor [2, 3]. Recently, the emergence of immunotherapy, especially the implementation of immune checkpoint inhibitors, has provided new hope for patients with advanced gastric cancer. Studies have revealed that blocking the signal transduction of programmed death receptor-1 (PD-1) and programmed death ligand 1 (PD-L1) can lead to sustained antitumour immunity [4]. Although PD-1/PD-L1 inhibitors have shown effectiveness for some patients, the majority of gastric cancer patients do not benefit from immunotherapy.

Solid tumours have a complex tumour microenvironment (TME), including tumour cells, endothelial cells, stromal cells and immune cells [5]. A hypoxic microenvironment is a widespread characteristic of solid tumours. Rapid growth of tumour cells results in insufficient blood supply to the tumour, resulting in a hypoxic TME [6, 7]. The hypoxic microenvironment can impair the ability of cytotoxic T cells and promote immune escape of cancer cells [8]. Additionally, the hypoxic microenvironment promotes the expression of PD-L1 on tumour cells [9–12]. High levels of PD-L1 are frequently linked to tumour immune evasion and progression. Overexpression of PD-L1 on cancer cells inhibits immune attack from CD8^+^ T cells through interaction with PD-1 [13].

Increasing evidence has shown that the hypoxic microenvironment can cause tumours to develop resistance to immune checkpoint blockade therapy by inhibiting antitumour immune effector cells and promoting immune escape. Research conducted on melanoma has demonstrated that tumours with hypoxic characteristics are associated with impaired T-cell function and resistance to anti-PD-1 (α-PD-1) therapy [14]. A previous study suggested that an increase in the oxidative metabolism of tumor cells results in higher intratumour hypoxia and a decrease in CD8^+^ T cells, ultimately leading to α-PD-1 resistance [15]. Additionally, nonresponders treated with α-PD-1 in gastrointestinal tumours exhibited upregulation of hypoxic metabolism-related signaling pathways [16]. Hence, it is imperative to explore an alternative approach to counteract the hypoxic microenvironment, reinstate the functionality and abundance of tumour-infiltrating T cells, and ultimately enhance the therapeutic efficacy of α-PD-1.

Nanotechnological carriers are especially useful for drugs that have low water solubility, as they can help to improve bioavailability and reduce potential adverse effects [17]. Nanomedicine-based approaches have been verified to enhance the systemic immune response and modulate the tumour microenvironment, including conversion from hypoxia to normoxia, transition from acidic to neutral conditions and transformation from nonimmunogenic to immunogenic states [18, 19]. Recent studies have shown that water-soluble catalase [20] or MnO_2_ NPs [21] carried by specific nanomaterials can directly generate oxygen in tumour tissue to alleviate tumour hypoxia. Although these approaches are efficient, their direct application in clinical practice remains challenging. Evidence suggests that normalizing tumour hypoxia through existing drugs is experimentally feasible and holds potential for clinical cancer treatment [22].

**Scheme 1:**
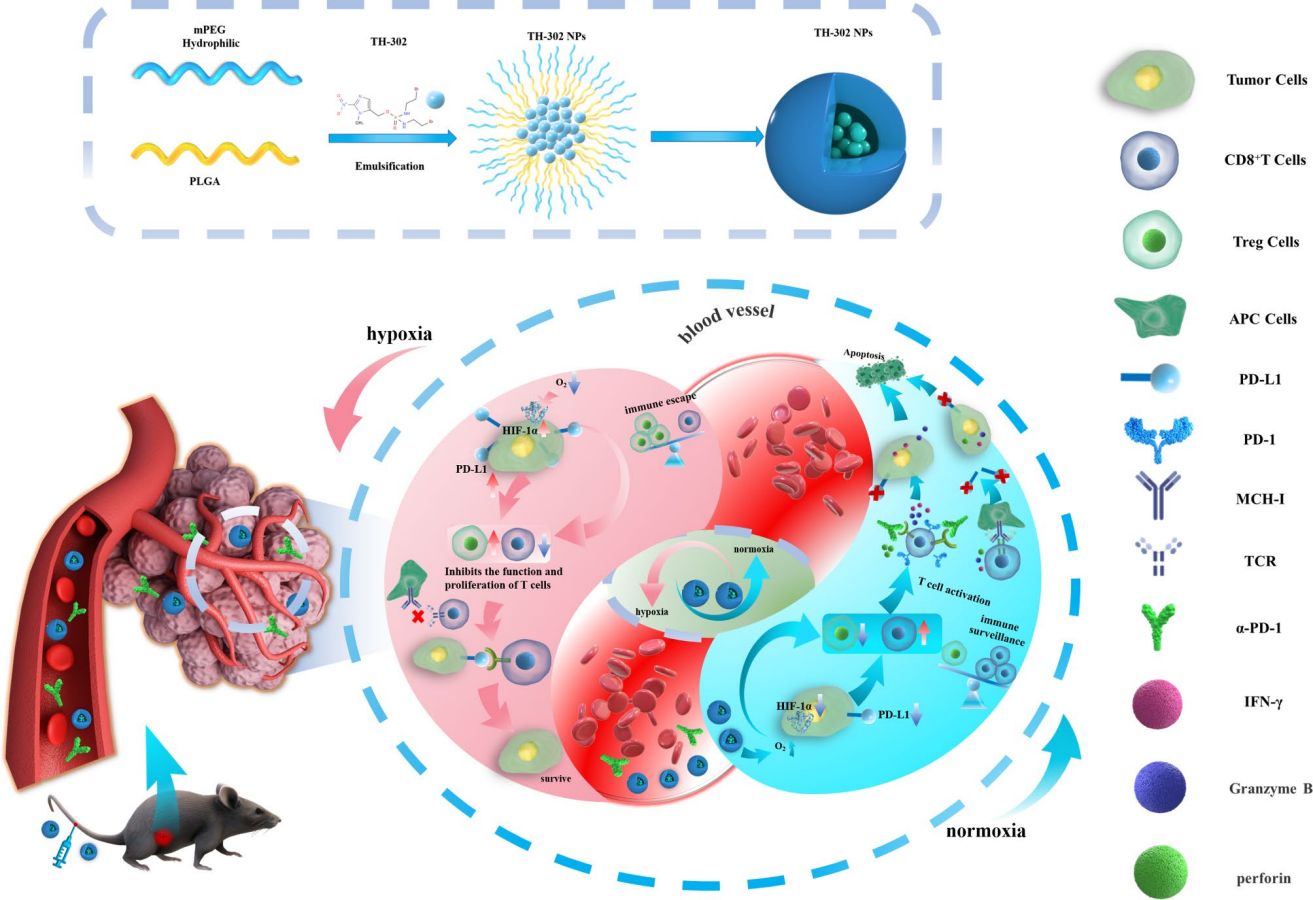
Schematic illustration of antitumour synergistic immunotherapy mediated by TH-302 NPs and α-PD-1 by alleviating the hypoxic tumour microenvironment

TH-302 is a hypoxia-activated prodrug that can be bioreduced to produce cytotoxic metabolites under hypoxic conditions [23]. Previous studies have shown that TH-302 can impede the growth of acute myeloid leukemia and nasopharyngeal carcinoma cells [24]. TH-302 can reduce the hypoxic area of prostate cancer and increase sensitivity to immune checkpoint blockers, and a decrease in hypoxia and normalization of the tumour vascular system is observed after TH-302 treatment [25]. Unlike other drugs that can ameliorate the hypoxic microenvironment, TH-302 has almost no adverse effect on normal tissues in the normoxic area [26]. However, TH-302 treatment in phase III clinical trials failed to show a statistically significant improvement in the overall survival of solid tumour patients, due to the inadequate accumulation of small-molecule anticancer drugs in tumour sites [27, 28]. To overcome these obstacles, we designed monomethoxy polyethylene glycol-Poly (lactic-co-glycolic acid) (mPEG-PLGA)-encapsulated TH-302 nanoparticles (TH-302 NPs) to enhance the bioavailability and tumour targeting of TH-302, thereby ameliorating the hypoxic tumour environment and enhancing the efficacy of α-PD-1 (Scheme 1).

## Methods

### Synthesis and preparation of TH-302 NPs

mPEG-PLGA polymer (mPEG-PLGA) and TH-302 NPs were prepared by the emulsion solvent evaporation method. To synthesize mPEG-PLGA bare nanoparticles with the desired particle size, 10 mg of mPEG-PLGA was dissolved in 0.5 mL of dichloromethane. This solution was slowly added to 4 mL of 1% Polyvinyl alcohol (PVA). Next, the mixture was phacoemulsified using a 100 W power ultrasonic switch with 4 s intervals for a total of 5 min. After that, the resulting microsphere suspension was stirred overnight using a magnetic stirrer to ensure complete volatilization of dichloromethane. The following day, the suspension was washed and centrifuged three times to obtain mPEG-PLGA nanoparticles with the desired size. TH-302 NPs were synthesized using a similar method. In brief, 20 mg of mPEG-PLGA and 0.5 mg of TH-302 were dissolved in 1 mL of dichloromethane solution at room temperature and refrigerated at 4 ℃ overnight. After mPEG-PLGA and TH-302 were fully dissolved, 1% PVA solution was slowly added and the mixture was emulsified through ultrasonic treatment. The emulsion was subsequently stirred continuously for 4 h through a magnetic stirrer to volatilize the dichloromethane in the solution and to solidify the particles. The sediment was collected through centrifugation at 12,000 rpm/min at 4 ℃ after evaporation. The collected sediment was then washed three times to obtain TH-302 NPs, which were subsequently resuspended in ddH_2_O.

### In vitro drug release

The drug release behavior of TH-302 NPs was investigated using the dynamic dialysis method [29, 30]. In brief, the TH-302 NPs suspension was placed in a dialysis bag and securely clamped at both ends. Subsequently, the bag was suspended in a brown bottle filled with 200 mL of phosphate-buffered saline (PBS) solution containing Tween 80. Samples were collected at the indicated time points, and the release medium was replenished accordingly. The concentration of TH-302 was determined by analyzing the peak area at 290 nm using high-performance liquid chromatography (Waters Corporation, USA) and calculating it based on the standard curve.

### Cellular uptake of NPs and lysosomal escape experiments

Fluorescent probe Coumarin-6 (C6) was used to detect the internalization of NPs by gastric cancer cells. MKN45 and MKN28 cells were treated with PBS, free C6 or mPEG-PLGA-C6 (C6 NPs) for 1 h. The concentration of C6 was 2 µg/mL. Then, the cells were fixed with 4% paraformaldehyde for 10 min, and the nuclei were stained with DAPI in dark for 5 min. The uptake of NPs in tumour cells was observed under a fluorescence microscope. In the lysosomal escape experiment, cells were treated as previously described and lysosomes were visualized using LysoTracker Red staining. Subsequently, qualitative analysis of the cells was conducted using confocal microscopy (Zeiss LSM 900, Germany).

### Cell spheroidization experiment

Serum-free medium was prepared with 1640/F12 medium, B27 supplement, recombinant epidermal growth factor and basic fibroblast growth factor. Gastric cancer cells were subsequently collected, washed with PBS, and suspended in serum-free medium. The cells were then inoculated into a 6-well plate with ultralow adhesion at a density of 1000 cells per well. TH-302 and TH-302 NPs were added as required for the experiment. The cultures were incubated for approximately 14 days, and microscope photos were taken for each group.

### In vivo biodistribution and tumor targeting of NPs

After the tumour model was successfully constructed, free DiR and mPEG-PLGA-DiR (DiR NPs) were injected through the tail vein. A small-animal live imaging system (AniView 100, China) was utilized to observe the fluorescence images of mice at 6 h, 24 h, 48 h, 72 h, and 120 h. Then, we repeated this animal experiment again. The tumours and major organs of the mice were removed at 48 h after injection and the ex vivo fluorescence intensity was measured.

### Immunofluorescence of hypoxic area in tumour tissues

One hour prior to euthanizing the animals, an intraperitoneal injection of 60 mg/kg of pimonidazole was administered. Tumour tissues were obtained and prepared for paraffin sectioning. Subsequently, FITC-MAb1 and HRP combined rabbit anti-FITC staining was performed for a duration of 1 h. Finally, the samples were photographed using confocal microscopy (Zeiss LSM 900, Germany).

### Extraction of human peripheral blood mononuclear cells (PBMCs)

Anticoagulant human peripheral blood was collected and diluted with PBS at a 1:1 ratio. The mixture was then mixed either by inversion or by pipetting. In a 15 mL centrifuge tube, 3 mL of thoroughly mixed Ficoll solution was added, followed by the careful addition of 2 mL of diluted blood along the tube wall. Successful separation of blood and Ficoll solution was observed. The tube was then centrifuged at 500 × g for 25 min. The upper yellow liquid was discarded, and the white thin-film layer in the middle, which consisted of single nuclear cells, was carefully absorbed. The obtained mononuclear cells were washed with 10 mL of PBS and centrifuged at 250 × g for 10 min, and then the supernatant was discarded. The cells were washed again and resuspended for future use.

### T-cell-mediated tumour cell killing test

PBMCs were cultured in RPMI-1640 medium and activated with human T activator CD3/CD28 for one week. MKN45 and MKN28 cells were seeded into 12-well plates. After 24 h, the activated PBMCs and adherent cells were cocultured for 48 h. After incubation for 48 h, cell fragments were removed and PBMCs were collected. Gastric cancer cells were then harvested and labelled with annexin V-FITC/PI for flow cytometry analysis.

### Tumourigenesis experiment in C57BL/6 mice

In this study, 2 × 10^6^ MFC cells were collected and resuspended in PBS. The cells were then subcutaneously inoculated into the groin at the left hind limb of C57BL/6 mice. Then the tumour-bearing mice were randomly divided into 6 groups: (1) control group (normal saline), (2) α-PD-1 group, (3) TH-302 group, (4) TH-302 NPs group, (5) TH-302 + α-PD-1 group, and (6) TH-302 NPs + α-PD-1 group. The corresponding drugs in each group were injected into mice twice a week. Tumour volume was monitored every three days using a Vernier calliper. Then the mice were sacrificed and tumour tissues were obtained. The tumour specimens were weighed and fixed for subsequent experiments.

### Biosafety evaluation of TH-302 NPs

BALB/c nude mice and C57BL/6 miouse models were utilized to assess the biological safety of TH-302 NPs. Tumours and major organs were collected from the animal models, followed by haematoxylin and eosin (H&E) measurements. The mouse serum levels of aspartate aminotransferase (AST), alanine aminotransferase (ALT), serum creatinine (CRE) and blood urea nitrogen (BUN) were examine to evaluate the liver and kidney function of the mice.

### Statistical analysis

GraphPad Prism 9.0 software was used to conduct statistical analyses. All values were expressed as the mean ± standard deviation (SD). The significance of differences among groups was determined using one-way ANOVA and Student’s t-test. Statistically significant is labeled as *P < 0.05, **P < 0.01, ***P < 0.001.

Supplementary Methods are described in Additional file 1.

## Results

### Preparation and characterization of TH-302 NPs

**Fig. 1:**
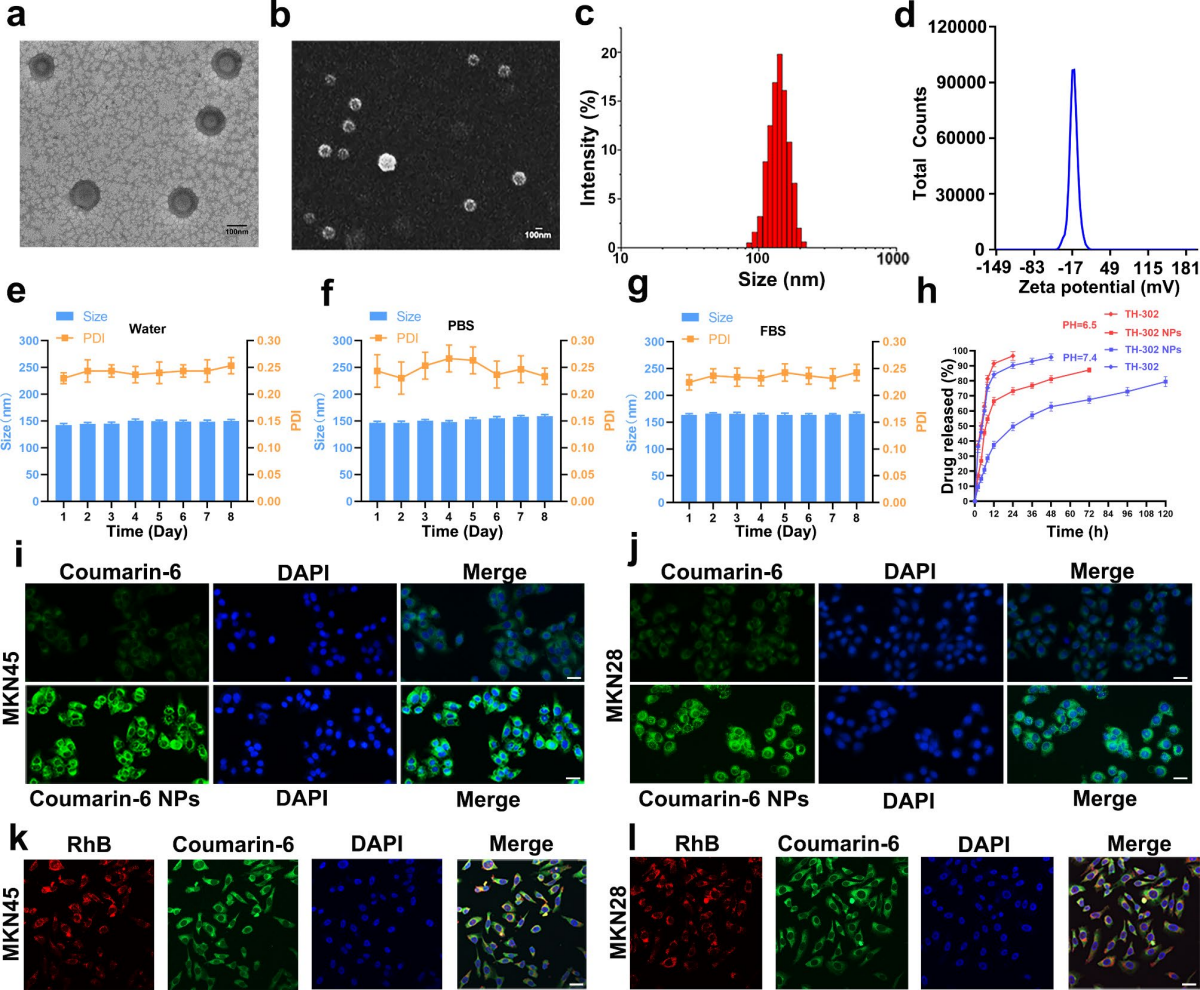
The preparation and characterization of TH-302 NPs. **a** Representative TEM images of TH-302 NPs. Scale bar: 100 nm. **b** Representative SEM images of TH-302 NPs. Scale bar: 100 nm. **c** The size distribution profile of the TH-302 NPs was measured using the dynamic light scattering method. **d** The zeta potential of TH-302 NPs. **e** Changes in the size and PDI of TH-302 NPs in water within 8 days. **f** Changes in the size and PDI of TH-302 NPs in PBS within 8 days. **g** Changes in the size and PDI of TH-302 NPs in 10% FBS within 8 days. **h** The drug release curve of TH-302 from TH-302 NPs in PBS buffer at different pH values. Detect the release of free TH-302 as a control. **i, j** MKN45 (**i**) and MKN28 (**j**) cells were incubated with C6 NPs or free C6 for 4 h, respectively. The fluorescent probe C6 was used to show the cellular uptake of mPEG-PLGA in vitro. Scale bar: 20 μm. **k**, **l** Colocalization of mPEG-PLGA-RhB and C6 in MKN45 (**k**) and MKN28 (**l**) cells in vitro. Scale bar: 20 μm

PLGA possesses distinctive drug delivery properties. The performance of PLGA NPs can be further enhanced by modifying their surface with PEG, which extends the circulation time of the NPs [31]. TH-302 NPs were prepared by the ultrasonic emulsification evaporation method. The morphology of TH-302 NPs was observed by transmission electron microscopy (TEM) and scanning electron microscopy (SEM) (Fig. 1a, b). The TEM and SEM results revealed that the NPs were uniformly sized, spherical, and well-dispersed without significant aggregation. The average particle size of TH-302 NPs was 140 ± 3.50 nm using the dynamic light scattering method (Fig. 1c). During the drying process, TH-302 NPs underwent shrinkage, resulting in a smaller average particle size detected by TEM than that detected using the dynamic light scattering method [31]. The zeta potential of TH-302 NPs was − 16.69 ± 1.60 mV (Fig. 1d). To assess the stability of TH-302 NPs, we measured the polydispersity index (PDI) and particle size of the NPs in water, PBS, and 10% Fetal bovine serum (FBS) over a period of 8 days. The results revealed that the PDI and particle size of the nanoparticles remained relatively unchanged in water and PBS (Fig. 1e, f), indicating their excellent stability in vitro. However, in the presence of 10% FBS, there was a slight increase in the diameter of TH-302 NPs, possibly due to the formation of protein coronas on their surface [32]. Nevertheless, the PDI of TH-302 NPs was minimally affected by the presence of FBS, suggesting that the surface of the NPs still maintained good stability even in serum (Fig. 1g). The encapsulation efficiency of TH-302 NPs was 68.20% ± 2.00%, and the loading efficiency was 1.72% ± 0.36%.

Drug release of TH-302 NPs in PBS buffer at different pH values was also investigated in our research. Our results indicated that approximately 40% of drugs are released within 24 h in PBS buffer with a pH of 7.4, whereas approximately 65% of drug release is observed in PBS buffer with a pH of 6.5 (Fig. 1h). These results indicated that the TH-302 NPs drug delivery system possessed the characteristic of controlled drug release. In vitro experiments were conducted to evaluate the uptake of mPEG-PLGA NPs by gastric cancer cells using C6. Compared to the free C6 group, coincubation of C6 NPs with MKN45 or MKN28 cells led to a significant increase in C6 fluorescence signal within the cytoplasm of gastric cancer cells in the C6 NPs group (Fig. 1i, j). These results indicated that mPEG-PLGA NPs could increase drug uptake by tumour cells. To demonstrate the encapsulation of C6 by nanoparticles, we employed mPEG-PLGA-RhB with a red fluorescent label to encapsulate C6. Subsequently, we observed the colocalization of red signal (indicating the presence of nanoparticles) and green signal (indicating the presence of C6) using a fluorescence microscope (Fig. 1k, l). These results confirmed that C6 could be transported into the cells by nanoparticles.

### Cellular internalization and endo/lysosomal Escape of TH-302 NPs for gastric cells

**Fig. 2:**
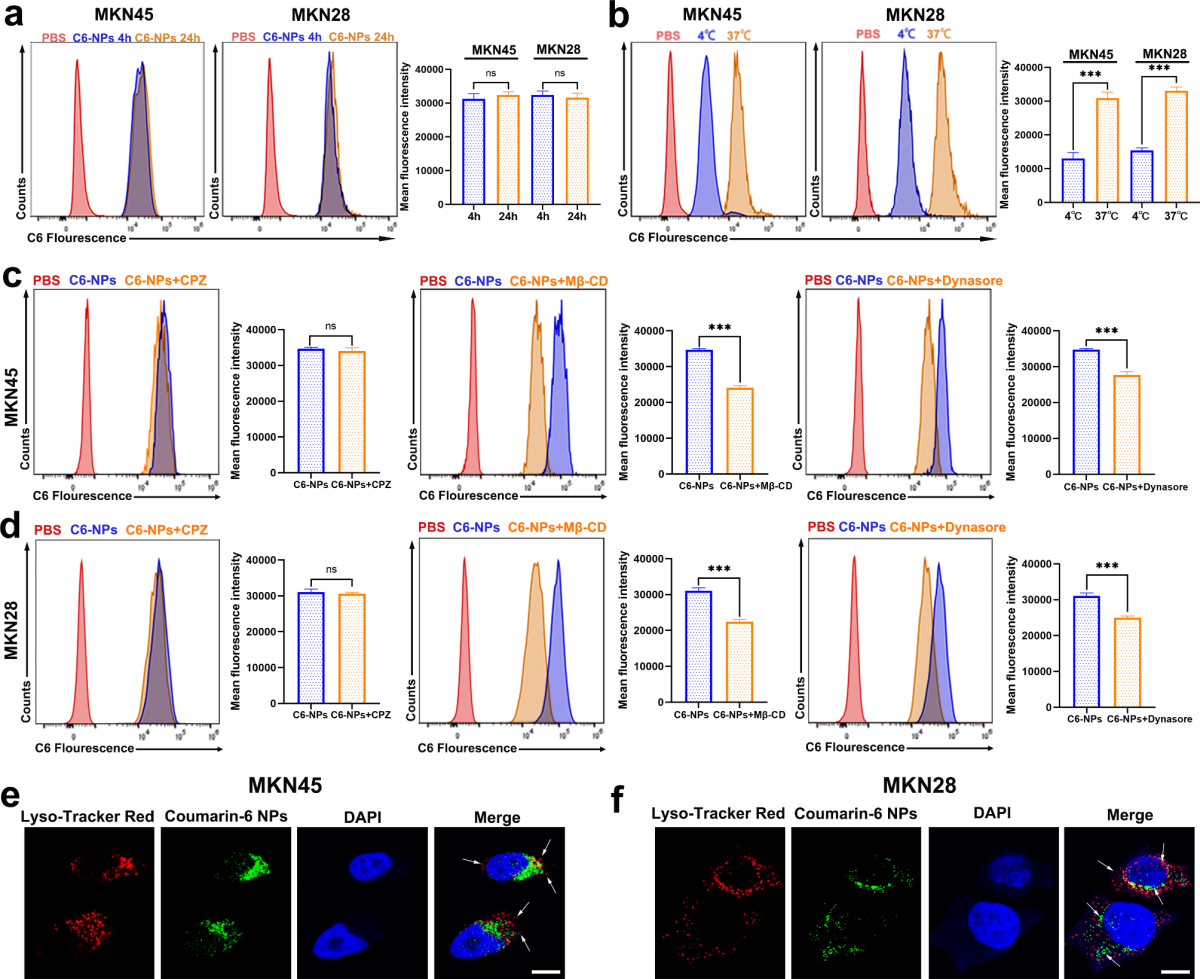
The cellular uptake of C6 NPs. **a** Cellular uptake of C6 NPs in MKN45 and MKN28 cells after 4 or 24 h of incubation measured by flow cytometry. **b** Cellular uptake of C6 NPs in MKN45 and MKN28 cells after incubation at 4 ℃ or 37 ℃. **c**, **d** Cellular uptake of C6 NPs in MKN45 (**c**) and MKN28 (**d**) cells treated with CPZ, Mβ-CD or dynasore. **e**, **f** Endo/lysosomal escape of C6 NPs in MKN45 (**e**) and MKN28 (**f**) cells. Cell nuclei were stained with DAPI (blue). Endolysosomes were stained with LysoTracker Red (red). Green fluorescence was from C6 NPs. Scale bar: 5 μm. Data are presented as mean ± SD. *** *P* < 0.001

After a 4-hour treatment, green fluorescence was observed in MKN28 and MKN45 cells loaded with C6 NPs. The intracellular C6 fluorescence signal remained similar at 24 h compared to 4 h, suggesting that uptake and internalization primarily occurred within the first 4 h (Fig. 2a). To investigate the cellular uptake mechanism of nanoparticles, we initially incubated C6 and C6 NPs with gastric cancer cells at both 4 ℃ and 37 ℃ for 4 h. We observed that cell endocytosis was inhibited at 4 ℃, indicating that the endocytosis of C6 nanoparticles is energy-dependent (Fig. 2b). We then conducted subsequent experiments to determine the pathway through which C6 NPs enter the cells. Prior to adding C6 NPs, the cells were preincubated with chlorpromazine (CPZ), methyl-β-cyclodextrin (Mβ-CD), and dynasore for 4 h. CPZ is an inhibitor of reticulin mediated endocytosis. Mβ-CD is a commonly used pharmacological inhibitor of caveolin-mediated endocytosis that can deplete cholesterol on the cell membrane to inhibit caveolin/lipid raft-dependent endocytosis. Dynasore can block the endocytic pathways mediated by reticulin and caveolin [33].The results showed no significant difference in the average fluorescence intensity of cells pretreated with CPZ compared to the control group, while the average fluorescence intensity of cells pretreated with Mβ-CD or dynasore was significantly lower than that in the control group (Fig. 2c, d). The above results indicate that the endocytosis of C6 NPs is related to caveolin and caveolin/lipid rafts. In addition, we used C6 NPs as models to investigate whether NPs can effectively escape from lysosomes. The results showed that C6 NPs and lysosomes had no colocalized fluorescence, indicating that the presence of C6 NPs promoted drug escape from lysosomes (Fig. 2e, f).

### TH-302 NPs could inhibit the proliferation of gastric cancer cells under hypoxic conditions

**Fig. 3:**
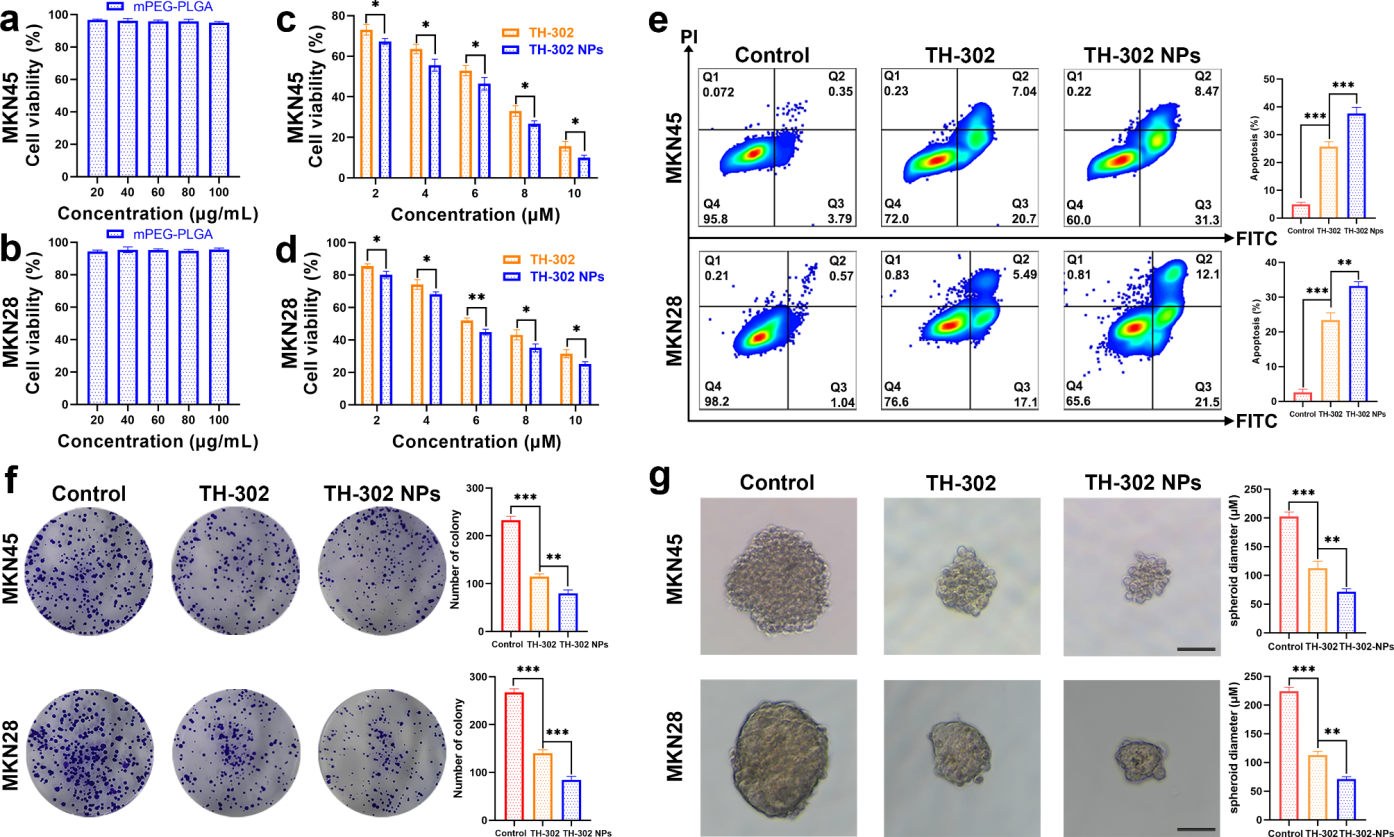
TH-302 NPs exhibited hypoxia-specific cytotoxicity. **a**, **b** mPEG-PLGA empty spheres have no toxic effect on MKN45 (**a**) and MKN28 (**b**) cells. **c**, **d** Toxic effects of TH-302 or TH-302 NPs on MKN45 (**c**) and MKN28 (**d**) cells in hypoxic environments. **e** Analysis of the apoptosis rate of MKN45 and MKN28 cells treated with TH-302 or TH-302 NPs under hypoxic conditions. **f** The effects of TH-302 or TH-302 NPs on the proliferative ability of MKN45 and MKN28 cells under hypoxic conditions. **g** The effects of TH-302 or TH-302 NPs on the proliferation of 3D gastric cancer cell spheroids. Scale bar: 50 μm. Data are presented as mean ± SD. *P < 0.05, **P < 0.01, ***P < 0.001

To evaluate the toxicity of TH-302 NPs to human gastric cancer cells (MKN45 and MKN28) in vitro, we performed a Cell Counting Kit-8 assay to measure cell viability. First, we evaluated the effectiveness of empty mPEG-PLGA vectors in inducing toxicity in MKN45 and MKN28 cells. The results indicated that mPEG-PLGA empty spheres had no significant cytotoxicity to gastric cancer cells (Fig. 3a, b). MKN45 and MKN28 cells were exposed to TH-302 and TH-302 NPs for 48 h under normoxic or hypoxic conditions. The results indicated that low concentrations of TH-302 had no significant cytotoxicity under normoxic conditions (Additional file 1: Fig. S1). However, under hypoxic conditions, low concentrations of TH-302 demonstrated significant cytotoxicity to gastric cancer cells (Fig. 3c, d). To investigate the antiproliferative effect of TH-302 on gastric cancer, we initially measured the apoptosis rates of MKN45 and MKN28 cells after different treatments. Our results showed that under hypoxic conditions, the TH-302 or TH-302 NPs groups had higher cell apoptosis rates than the control group (Fig. 3e). When exposed to normoxic conditions, gastric cancer cells did not undergo significant apoptosis at low concentrations of TH-302 and TH-302 NPs (Additional file 1: Fig. S2). The results of the colony formation assay revealed that treatment with low concentrations of TH-302 NPs significantly decreased the proliferation of gastric cancer cells under hypoxic conditions, thus confirming the hypoxic selective cytotoxicity of TH-302 NPs (Fig. 3f). We performed validation experiments using tumour 3D cell spheroids. When exposed to hypoxic environments, TH-302 and TH-302 NPs both demonstrated a significant inhibitory effect on the growth of tumour spheroids (Fig. 3g).

### TH-302 could effectively downregulate the expression of HIF-1α and PD-L1 in gastric cancer cells

**Fig. 4:**
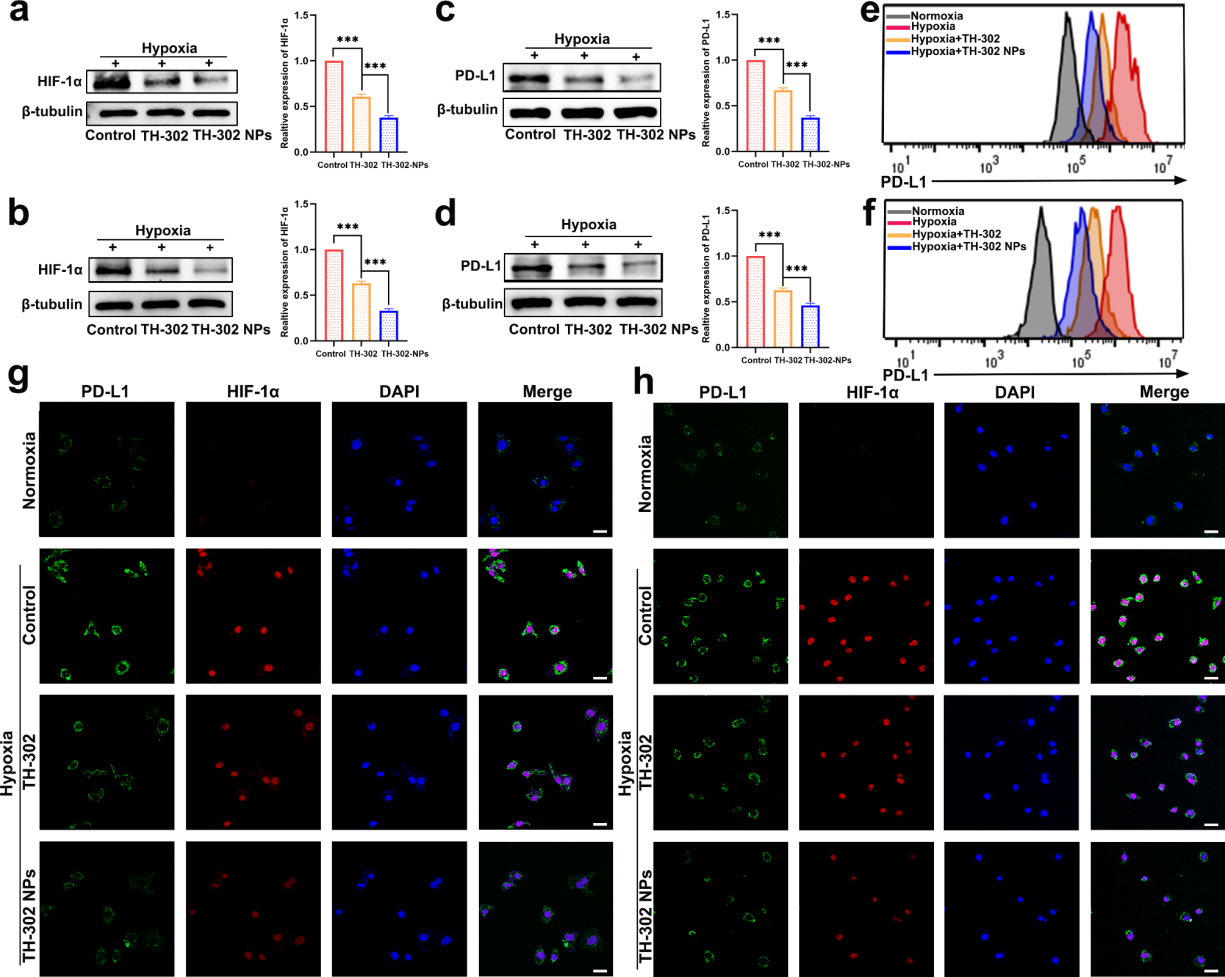
TH-302 NPs downregulated the expression of HIF-1αand PD-L1 in tumour cells. **a**, **b** The protein levels of HIF-1α in MKN45 (**a**) and MKN28 (**b**) cells treated with TH-302 or TH-302 NPs were examined using western blot analysis. **c**, **d** The protein levels of PD-L1 in MKN45 (**c**) and MKN28 (**d**) cells treated with TH-302 or TH-302 NPs were assessed by western blot analysis. **e**, **f** Analysis of PD-L1 expression in MKN45 (**e**) and MKN28 (**f**) cells using flow cytometry. **g**, **h** Immunofluorescence staining of PD-L1 and HIF-1α in MKN45 (**g**) and MKN28 (**h**) cells treated with TH-302 or TH-302 NPs. Scale bars: 20 μm. Data are presented as mean ± SD. ***P < 0.001

MKN45 and MKN28 cells were cultured in hypoxic or normoxic environments for 48 h. The cells were then collected, and proteins were extracted for western blot experiments. We observed that HIF-1α was not expressed in MKN45 and MKN28 cells under normoxic conditions. However, there was a significant increase in the expression of HIF-1α in both MKN45 and MKN28 cells after hypoxia induction (Additional file 1: Fig. S3). Subsequent to hypoxia induction, gastric cancer cells were treated with TH-302 or TH-302 NPs. The western blot results indicated a significant reduction in the levels of HIF-1α in gastric cancer cells upon treatment with TH-302 and TH-302 NPs (Fig. 4a, b). The expression levels of PD-L1 in gastric cancer cells in different groups was detected by flow cytometry and western blot assays. The results indicated that exposure to hypoxic conditions led to upregulation of PD-L1 in MKN45 and MKN28 cells. Notably, treatment with TH-302 and TH-302 NPs effectively reversed this trend (Fig. 4c-f). Confocal fluorescence microscopy was used to examine the expression levels of PD-L1 and HIF-1α in gastric cancer cells in different groups. The fluorescence intensity of PD-L1 and HIF-1α was enhanced in hypoxic environments and significantly decreased after the addition of TH-302 or TH-302 NPs (Fig. 4g, h). These findings were consistent with the western blot results.

### Antitumour activity of TH-302 NPs in a gastric cancer xenograft tumour model

**Fig. 5:**
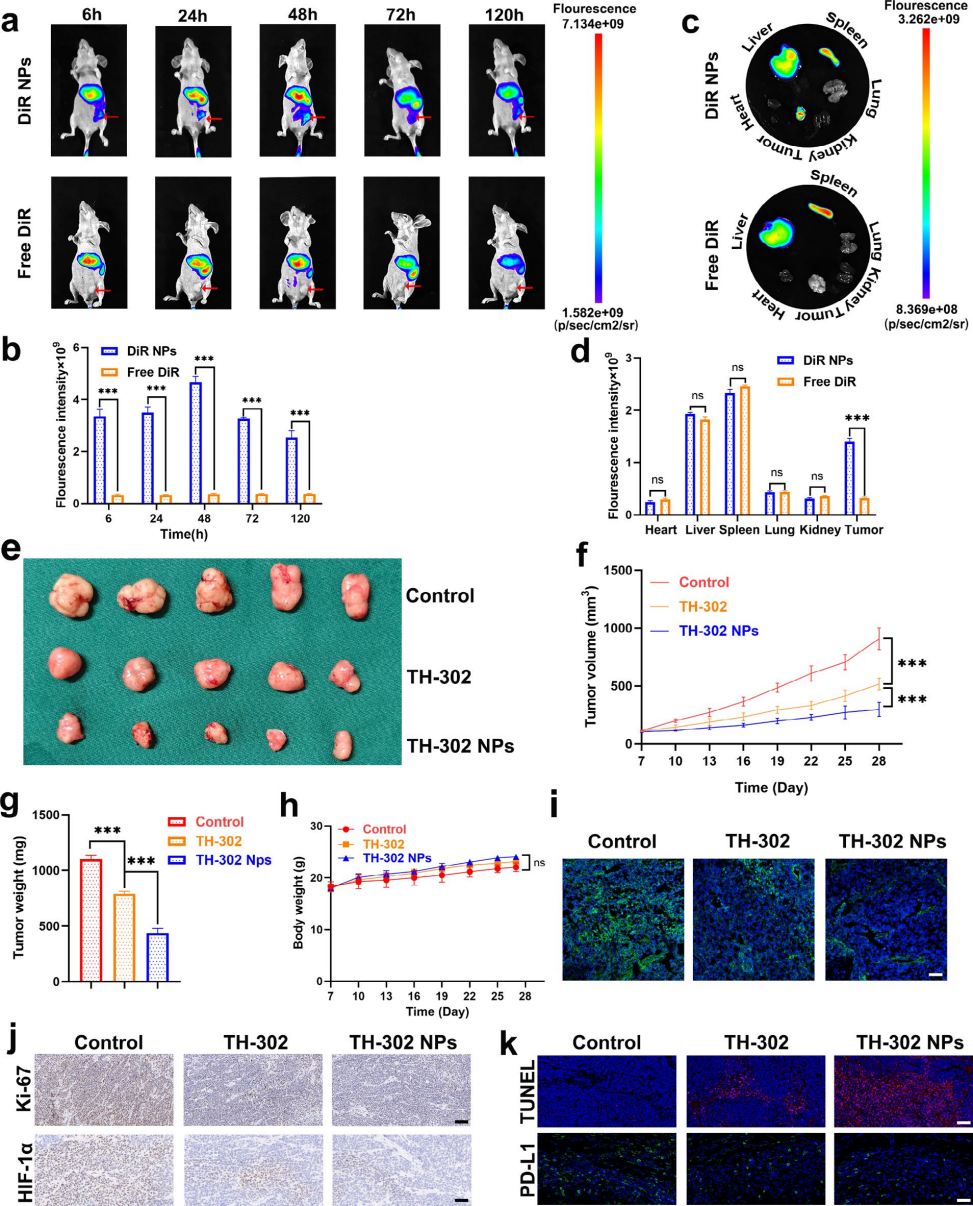
Evaluation of tumour targeting and antitumour effects of TH-302 NPs in vivo. **a**, **b** Real-time observation of the distribution of DiR NPs in BALB/c nude mice using small-animal live imaging system. A comparative statistical chart of fluorescence signal intensity is shown. **c**, **d** The ex vivo fluorescence images of tumours and major organs after injection with DiR NPs or free DiR. A comparative statistical chart of fluorescence signal intensity is shown. **e** Photographs of the subcutaneous tumours (n = 5 for each group). **f** Subcutaneous tumour volume growth curves are depicted. **g** The tumour weight were measured. **h** The weight of the mice among the three groups. **i** Immunofluorescence staining using pimonidazole for tumour tissue sections. Scale bar: 100 μm. **j** Immunohistochemistry of Ki-67 and HIF-1α in subcutaneous tumour tissue sections. Scale bar: 100 μm. **k** Immunofluorescence staining of TUNEL and PD-L1 in subcutaneous tumour tissue sections. Scale bar: 100 μm. Data are presented as mean ± SD. **P < 0.01, ***P < 0.001

After confirming the antitumour effect of TH-302 NPs on gastric cancer cells in vitro, we then established gastric cancer subcutaneous tumours in BALB/c nude mice. To observe the biological distribution of TH-302 NPs in vivo, we utilized a small-animal live imaging system to monitor the real-time distribution of DiR NPs. After injecting DiR NPs into the tail vein of mice, the fluorescence signal in the subcutaneous tumour area of the left groin increased and peaked at 48 h (Fig. 5a, b). No fluorescence accumulation was observed in the tumour area of the free DiR group within 120 h. Ex vivo fluorescence images of the tumours and organs also confirmed these results, demonstrating the excellent tumour-targeting ability of DiR NPs (Fig. 5c, d). The results demonstrated that the control group exhibited the highest tumour volume and weight. However, the treatment group, which utilized TH-302 or TH-302 NPs, exhibited a significant reduction in tumour volume and weight. Notably, the therapeutic efficacy of the TH-302 NPs is higher than TH-302 alone (Fig. 5e-g). This phenomenon could be attributed to the tumour targeting and in vivo sustained-release properties of TH-302 NPs. Among the three groups, there was no significant difference in the body weight of the mice, indicating that the toxicity of TH-302 NPs was negligible (Fig. 5h). Pimonidazole is commonly used to reflect the hypoxic level within tumour tissues [34, 35]. As shown in Fig. 5i, the hypoxic area of tumour tissues was significantly decreased after TH-302 and TH-302 NPs treatment. Additionally, Ki-67 staining of tumour tissue sections indicated that the TH-302 NPs group had the lowest level of tumour proliferation. TUNEL staining revealed that the TH-302 NPs group exhibited the highest rate of cell apoptosis, which was consistent with the in vitro results. We also investigated the expression levels of HIF-1α and PD-L1 in tumour tissues in different groups. The results showed that decreased expression levels of HIF-1α and PD-L1 were found in the tumour tissues after treatment with TH-302 and TH-302 NPs (Fig. 5j, k). These findings demonstrated that TH-302 NPs exhibited excellent antitumour activities in vivo.

### TH-302 NPs could reverse T-cell incompetence under hypoxic conditions and enhance the efficacy of α-PD-1 therapy in vitro

**Fig. 6:**
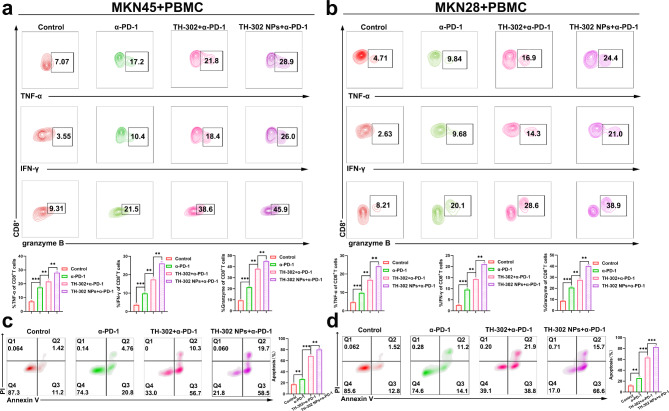
TH-302 NPs could reverse T-cell incompetence under hypoxic conditions and enhance the efficacy of α-PD-1 therapy. **a, b** Expressions of TNF-α, IFN-γ and granzyme B in different groups of MKN45 (**a**) and MKN28 (**b**) cells under hypoxic conditions. **c, d** Representative images of the flow cytometry analysis of the PBMCs-mediated elimination of MKN45 (**c**) and MKN28 (**d**) cells, as determined by the annexin V-FITC/PI double labelling method. Data are presented as mean ± SD. **P < 0.01, ***P < 0.001

We cocultured activated PBMCs and gastric cancer cells under normoxic and hypoxic conditions for 48 h. Initially, we assessed the proportion of CD3^+^CD8^+^ T cells in PBMCs under normoxic and hypoxic conditions. The results revealed a significant reduction in the proportion of CD3^+^CD8^+^ cells in hypoxic environments (Additional file 1: Fig. S4), suggesting a substantial inhibition of cytotoxic T lymphocytes (CTLs) growth. Subsequently, we treated the cocultured cells with the corresponding drugs in different groups. Compared to the α-PD-1 group, the combination of TH-302 or TH-302 NPs with α-PD-1 did not alter the levels of TNF-α, IFN-γ, and granzyme B in normoxic environments (Additional file 1: Fig. S5a, b). However, in hypoxic environments, the combination of TH-302 NPs with α-PD-1 significantly increased cytokine secretion by CTLs (Fig. 6a, b). The results showed that TH-302-NPs could effectively enhance the efficacy of α-PD-1 and bolster the antitumour function of cytotoxic T cells in hypoxic environments. Moreover, we employed flow cytometry to analyze the apoptosis rate of gastric cells in each group. Our findings demonstrated that the coadministration of TH-302 NPs and α-PD-1 exerted a stronger antitumour effect by enhancing the effectiveness of cytotoxic T cells in eradicating tumours (Fig. 6c, d).

### TH-302 NPs enhanced the efficacy of α-PD-1 immunotherapy in vivo

**Fig. 7:**
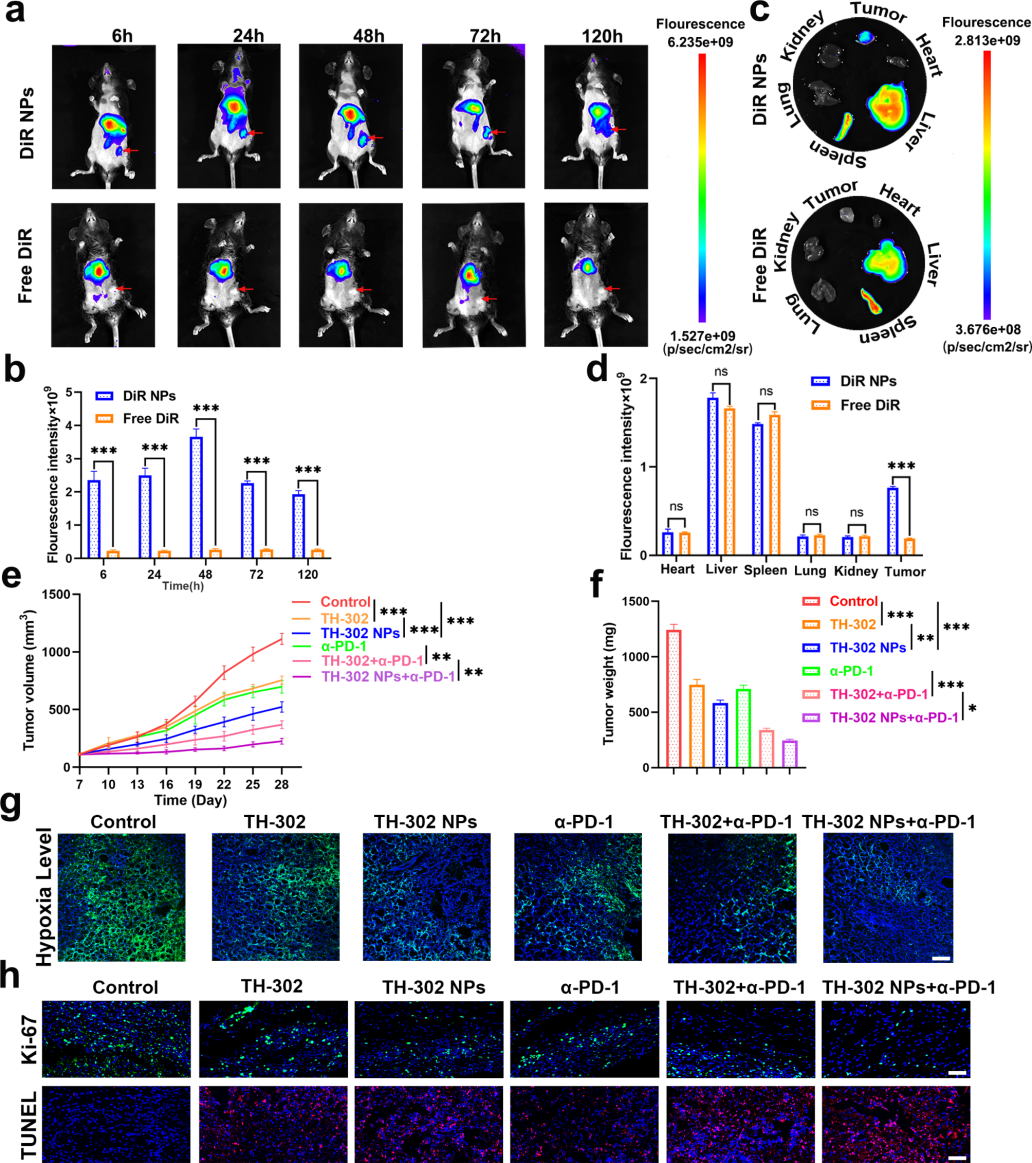
TH-302 NPs enhanced the efficacy of PD-1 blockade immunotherapy in vivo. **a**, **b** Real-time observation of the distribution of DiR NPs in C57BL/6 mice using small-animal live imaging system. A comparative statistical chart of fluorescence signal intensity is shown. **c**, **d** The ex vivo fluorescence images of tumours and major organs after injection with DiR NPs or free DiR. A comparative statistical chart of fluorescence signal intensity is shown. **e** The tumour volume growth curves are depicted (n = 5 for each group). **f** The tumour weight were measured at the indicated time points. **g** Immunofluorescence staging using pimonidazole for tumour tissue sections. Scale bar: 100 μm. **h** Ki-67 staining and TUNEL analysis of the indicated xenograft tumour samples. Scale bar: 100 μm. Data are presented as mean ± SD. *P < 0.05, **P < 0.01, ***P < 0.001

We attempted to investigate whether TH-302 NPs could enhance the efficacy of α-PD-1 by alleviating the hypoxic tumour microenvironment. The homograft mouse models were established by subcutaneously injecting of MFC cells. To observe the distribution of TH-302 NPs in vivo, we employed DiR as a model drug to examine the biological distribution and monitored the distribution using a small-animal live imaging system in real-time. The free DiR group did not show any accumulation of fluorescence signal in the tumour area within 120 h. However, the fluorescence signal in the tumour area was significantly enhanced in the DiR NPs group and reached its peak at 48 h (Fig. 7a, b). The tumour-targeting ability of DiR NPs was also confirmed by ex vivo fluorescence images of tumours and organs. A higher tumour fluorescence signal intensity was found in the DiR NPs group than that in the Free DiR group, providing evidence that the encapsulation of NPs enhanced the ability of drugs to target tumour tissues (Fig. 7c, d). As shown in Fig. 7e and f, we found that the combination of TH-302 NPs with α-PD-1 exhibited a synergistic antitumour effect, which was superior to that of the other groups. Notably, the TH-302 NPs group exhibited better therapeutic effects than the TH-302 group, consistent with the abovementioned results in the BALB/c nude mouse model. We used pimonidazole staining to evaluate the tumour hypoxia area of each group. When combined with α-PD-1 treatment, TH-302 NPs significantly reduced the hypoxic area of tumour tissues (Fig. 7g). Additionally, the level of Ki-67 in tumour tissues was the lowest in the TH-302 NPs + α-PD-1 group. The proportion of TUNEL positive cells in tumours treated with combined therapy was significantly higher than that in tumours treated with α-PD-1 or TH-302 NPs alone (Fig. 7h). Collectively, these results indicated that TH-302 NPs could alleviate the hypoxic tumour microenvironment and greatly improve the efficacy of PD-1 blockade.

### TH-302 NPs promoted CD8^+^ T-cell infiltration in tumour tissues in vivo

**Fig. 8:**
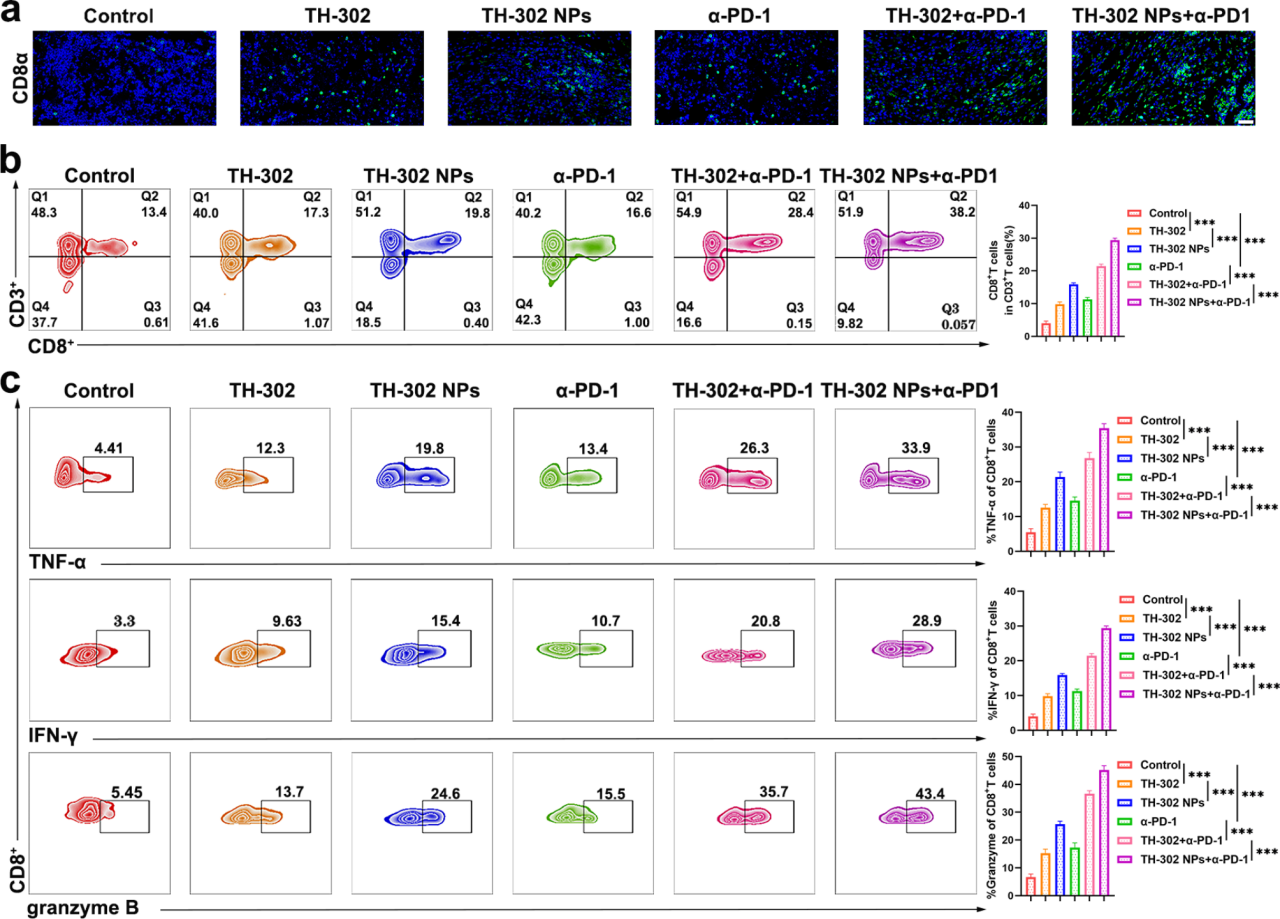
TH-302 NPs promoted CD8^+^ T-cell infiltration in tumour tissues in vivo. **a** Representative image of CD8α staining in the different treatment groups. Scale bar: 100 μm. **b** Flow cytometry analysis of CD3^+^CD8^+^ cells in tumours from the different treatment groups. **c** Representative images of the flow cytometry analysis of TNF-α^+^, IFN-γ^+^, and granzyme B^+^ CD8^+^ TILs from the six groups. Data are presented as mean ± SD. ***P < 0.001

To investigate the potential mechanism by which TH-302 NPs enhance PD-1 blockade immunotherapy, we analyzed the presence of CTLs within xenograft tumours. CTLs, particularly the CD8^+^ T cell subtype, play a crucial role in eliminating tumour cells [36]. Immunofluorescence analysis of tumour tissues revealed that the combination of TH-302 NPs with α-PD-1 treatment resulted in the highest infiltration of CD8^+^ T cells among the six groups (Fig. 8a). We obtained single-cell suspensions of tumor-infiltrating lymphocytes (TILs) from tumor tissues for flow cytometry analysis. The results revealed that the tumours treated with TH-302 NPs and α-PD-1 exhibited a significantly higher infiltration of CD8^+^ T cells than the other groups (Fig. 8b). Furthermore, the combination of TH-302 NPs with α-PD-1 significantly promoted the levels of TNF-α, IFN-γ, and granzyme B in tumour tissues (Fig. 8c). These results suggested that TH-302 NPs could enhance the infiltration of T lymphocytes in tumour tissues by alleviating the tumour immunosuppressive microenvironment in vivo, leading to an improved efficacy of immunotherapy.

### TH-302 NPs maintain a highly biocompatibility and biosafety

**Fig. 9:**
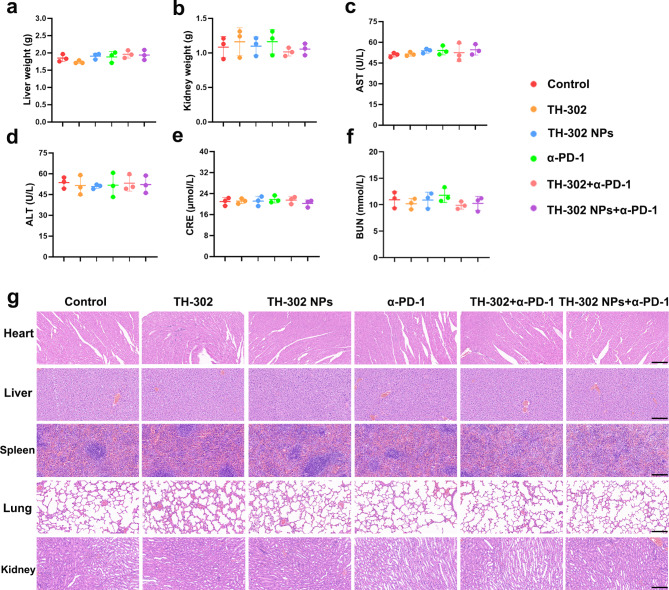
Biosafety evaluation of TH-302 NPs in vivo. **a** The liver weight of the C57BL/6 mice in the different treatment groups. **b** The kidney weight of the C57BL/6 mice in the different treatment groups. **c-f** The levels of serum AST (**c**), ALT (**d**), CRE (**e**)and BUN (**f**) in the C57BL/6 mice in the different treatment groups. **g** Representative images of mouse heart, liver, spleen, lung, and kidney sections stained with H&E staining. Scale bar: 100 μm. Data are presented as mean ± SD

Although the raw materials used in the present study have been approved by the Food and Drug Administration (FDA), it is still necessary to determine the biological safety of TH-302 NPs. Thus, we investigated the biosafety of TH-302 NPs in major organs, such as the heart, liver, spleen, lung and kidney of BALB/c nude mice and C57BL/6 mice. Our results indicated that TH-302 NPs did not affect the weight of the liver (Fig. 9a) and kidney (Fig. 9b) of C57BL/6 mice when compared to the control group. There were no significant differences in the levels of serum ALT, AST, CRE, and BUN of the mice among all the groups (Fig. 9c-f, Additional file 1: Fig. S6a-d). The H&E staining results indicated that TH-302 NPs did not exhibit significant systemic toxicity in BALB/c nude mice (Additional file 1: Fig. S6e) and C57BL/6 mice (Fig. 9g) compared to the control group. These findings suggested that TH-302 NPs exhibited high biocompatibility in vivo.

## Discussion

Immune checkpoint blockade led to a significant increase in T-cell infiltration in the normoxic regions of the tumour. However, only a few of these cells were able to reach the hypoxic areas of the cancer [25]. By reversing the immune-deficient tumour microenvironment, it is possible to restore tumour sensitivity to systemic α-PD-1 therapy. Hypoxia acts as a major obstacle for T-cell infiltration in tumour. Alleviation of the hypoxic microenvironment can greatly enhance sensitivity to immunotherapy, activate antitumour immune responses and facilitate tumour regression [37]. Our study highlighted the significance of the hypoxic tumour microenvironment in cancer immunotherapy and aimed to explore the effectiveness of TH-302 NPs in alleviating hypoxic microenvironment and enhancing the efficacy of α-PD-1 therapy in gastric cancer.

TH-302, a hypoxia-activating prodrug, has exhibited the ability to reduce the hypoxic microenvironment of tumour tissues in nasopharyngeal carcinoma and prostate cancer [24, 25, 38]. Additionally, it has shown effective antitumour properties in a hypoxic microenvironment without any observed toxic effects on tissues with normal oxygen content [39]. The unique dual effect of TH-302 sparked our curiosity about its potential effectiveness when used in combination with α-PD-1 treatment. However, the phase III clinical trial of TH-302 combined with gemcitabine in the treatment of patients with advanced pancreatic cancer missed the endpoint [40]. This study revealed that the drug was unable to effectively target tumours over an extended period, indicating that TH-302 alone might not be a reliable antitumour medication.

PLGA is a polymer compound synthesized from lactic acid and hydroxy lactic acid and approved by the FDA for human biomedical applications due to its biocompatibility, degradability, and nontoxic properties [41]. mPEG is widely used in drug delivery applications due to its ability to modify drugs and improve their efficacy. The FDA has classified mPEG as “generally recognized as safe”, making it a popular choice for pharmaceutical corporations [42]. An increasing number of mPEG-PLGA copolymers have been developed with mPEG serving as the hydrophilic shell and PLGA as the bone substitute structure. These copolymers have been utilized to administer hydrophobic drugs for the treatment of various diseases [43].

mPEG-PLGA, an amphiphilic copolymer, has garnered significant attention for its ability to self-assemble into NPs in aqueous solutions [44]. It is commonly used with various hydrophobic drugs or genes. These NPs possess a core-shell structure, where the drugs or genes reside in the core and are surrounded by hydrophilic shells. This configuration enables prolonged blood circulation and reduces uptake by the liver and spleen. Moreover, owing to the enhanced permeability and retention effect in solid tumour tissue, aggregated NPs tend to accumulate in tumour tissues and gradually degrade, resulting in the slow release of drugs [42, 45]. These characteristics of mPEG-PLGA have sparked our interest in using it to encapsulate TH-302 and enhance its aggregation in tumour tissue.

In this study, a nanodrug delivery system loaded with TH-302 was constructed using mPEG-PLGA. We demonstrated that TH-302 NPs possess uniform size, spherical shape, and excellent dispersion, with no noticeable aggregation. Moreover, these nanoparticles exhibit remarkable stability in water, PBS, and FBS. These characteristics were favorable for the aggregation of TH-302 NPs in tumour tissues [46, 47]. In vitro experiments showed that treatment with TH-302 NPs under hypoxic conditions significantly reduced the expression of PD-L1 and HIF-1α in gastric cancer cells. These findings indicated a potential therapeutic strategy by combining TH-302 NPs with immunotherapy for gastric cancer. As cytotoxic T cells, CD8^+^ T cells play a critical role in eliminating tumour cells [48]. In immune-competent mice, a substantial increase in the infiltration of CD8^+^ T cells within tumour tissues was observed after treatment with TH-302 NPs. Additionally, the levels of cytokines associated with immune cell activity significantly rose following tumour immunosuppressive microenvironment remission. This alleviation of immune suppression notably enhances the efficacy of α-PD-1. Most importantly, the unchanged blood biochemical indicators and H&E staining of the major organs of mice provided evidence for the biological safety of TH-302 NPs.

TH-302 NPs were confirmed to have high biocompatibility and tumour-targeting ability. However, this system can be improved through an N-(3-Dimethylaminopropyl)-N′-ethylcarbodiimide hydrochloride/N-hydroxysuccinimide-mediated amide reaction by directly binding immune checkpoint inhibitors to PLGA, which enhances immune system function and combats cancer [49]. Another option is to select appropriate targeting ligands, such as proteins, nucleic acid aptamers, peptides, sugars, and polysaccharides, to modify the surface of NPs and actively target cancer cells [50, 51]. Furthermore, other relevant materials or carriers, such as liposomes, may also be used to encapsulate TH-302 and reduce the hypoxic tumour microenvironment. More advanced nanomaterials will be further investigated in our future studies.

## Conclusions

In summary, the present study indicated that TH-302 NPs could reduce the expressions of PD-L1 and HIF-1α, promote CD8^+^ T-cell infiltration in tumour tissues and enhance the efficacy of α-PD-1 immunotherapy by alleviating the hypoxic microenvironment. Moreover, TH-302 NPs exhibited high bioavailability, effective tumour-targeting ability and satisfactory biosafety, thereby providing a potential strategy for combined immunotherapy in gastric cancer treatment.

### Electronic supplementary material

Below is the link to the electronic supplementary material.

**Additional file 1**: Fig. S1. TH-302 and TH-302 NPs have no cytotoxicity to MKN45 (a) and MKN28 (b) cells under normoxic conditions. Fig. S2. The Effects of TH-302 NPs on gastric cancer cells apoptosis under normoxic conditions. Fig. S3. The expression of HIF-1α in MKN45 and MKN28 cells under hypoxic and normoxic conditions. Fig. S4. Hypoxia inhibits the proportion of CD3^+^CD8^+^ cells in PBMCs. Fig. S5. The effects of TH-302 NPs on cytokine expression secreted by PBMCs cocultured with MKN45 (a) and MKN28 (b) cells under normoxic conditions. Fig. S6. Biosafety evaluation of TH-302 NPs in BALB/c nude mice. a-d The levels of serum AST (a), ALT (b), CRE (c)and BUN (d) in the BALB/c nude mice in the different treatment groups. e Representative images of mouse heart, liver, spleen, lung, and kidney sections of BALB/c nude mice stained with H&E staining. Scale bar: 100 μm. Data are presented as mean ± SD.

## Data Availability

All data generated or analyzed during this study are included in this published article and its supplementary information files.

## Abbreviations

PD-1: programmed death receptor-1
PD-L1: programmed death ligand 1
NPs: nanoparticles
TME: tumour microenvironment
SEM: scanning electron microscopy
α-PD-1: anti-PD-1
mPEG: monomethoxy polyethylene glycol
PLGA: poly (lactic-co-glycolic acid)
PVA: poly vinyl alcohol
C6: coumarin-6
C6 NPs: mPEG-PLGA-C6
DiR NPs: mPEG-PLGA-DiR
PBMCs: human peripheral blood mononuclear cells
H&E: hematoxylin and eosin
AST: aspartate transaminase
ALT: alanine transaminase
CRE: serum creatinine
BUN: blood urea nitrogen
TH-302 NPs: mPEG-PLGA-TH-302
FBS: fetal bovine serum
PBS: phosphate-buffered solution
PDI: polydispersity index
CPZ: chlorpromazine
Mβ-CD: methyl-β-cyclodextrin
CTLs: cytotoxic T lymphocytes
TILs: tumour-infiltrating lymphocytes
FDA: Food and Drug Administration
