# Supplementary material for: TH-302-loaded nanodrug reshapes the hypoxic tumour microenvironment and enhances PD-1 blockade efficacy in gastric cancer
Source: J Nanobiotechnology. 2023 Nov 22;21:440. doi: 10.1186/s12951-023-02203-8 (PMC10664313; doi:10.1186/s12951-023-02203-8)
Supplement: Supplementary file 1 — Additional file 1: Fig. S1. TH-302 and TH-302 NPs have no cytotoxicity to MKN45 (a) and MKN28 (b) cells under normoxic conditions. Fig. S2. The Effects of TH-302 NPs on gastric cancer cells apoptosis under normoxic conditions. Fig. S3. The expression of HIF-1α in MKN45 and MKN28 cells under hypoxic and normoxic conditions. Fig. S4. Hypoxia inhibits the proportion of CD3+CD8+ cells in PBMCs. Fig. S5. The effects of TH-302 NPs on cytokine expression secreted by PBMCs cocultured with MKN45 (a) and MKN28 (b) cells under normoxic conditions. Fig. S6. Biosafety evaluation of TH-302 NPs in BALB/c nude mice. a-d The levels of serum AST (a), ALT (b), CRE (c)and BUN (d) in the BALB/c nude mice in the different treatment groups. e Representative images of mouse heart, liver, spleen, lung, and kidney sections of BALB/c nude mice stained with H&E staining. Scale bar: 100 μm. Data are presented as mean ± SD. [file 12951_2023_2203_MOESM1_ESM.docx]

**Additional file information**

**TH-302-loaded nanodrug reshapes the hypoxic tumour microenvironment and enhances PD-1 blockade efficacy in gastric cancer**

Zhixiong Wang^1†^, Menglin Zhu^1†^, Runyu Dong^1^, Danping Cao^1^, Yanna Li^1^, Zhiqiang Chen^2*^, Juan Cai^3,4*^, and Xueliang Zuo^1,3*^

^1^Department of Gastrointestinal Surgery, The First Affiliated Hospital, Yijishan Hospital of Wannan Medical College, Wuhu 241001, China.

^2^Hepatobiliary Center, The First Affiliated Hospital of Nanjing Medical University, Key Laboratory of Liver Transplantation, Chinese Academy of Medical Sciences, NHC Key Laboratory of Liver Transplantation, Nanjing 210029, China.

^3^Anhui Province Key Laboratory of Non-coding RNA Basic and Clinical Transformation, Wannan Medical College, Wuhu 241001, China.

^4^Department of Oncology, The First Affiliated Hospital, Yijishan Hospital of Wannan Medical College, Wuhu 241001, China.

^†^Zhixiong Wang and Menglin Zhu contributed equally to this work.

* Correspondence:

Xueliang Zuo

zuoxueliang0202@126.com

Juan Cai

caijuan1987@yeah.net

Zhiqiang Chen

zqchen@njmu.edu.cn

**Supplementary Methods**

**Reagents**

mPEG-PLGA copolymer was purchased from Jinan Daigang Bioengineering (Shandong, China). mPEG-PLGA-RhB copolymer was obtained from Xi’an Qiyue Bioengineering (Shaanxi, China). Fetal bovine serum (FBS), phosphate-buffered saline (PBS) and RPMI-1640 medium were obtained from Thermo Fisher Scientific (Waltham, Massachusetts, USA). TH-302, coumarin-6, DiR and dichloromethane were purchased from Aladdin (Shanghai, China). Polyvinyl alcohol (PVA) and Annexin V-FITC/PI double staining kits were acquired from Sigma-Aldrich (St. Louis, Missouri, USA). A lysosomal staining kit (red fluorescence), an ECL chemiluminescence reagent kit, RIPA lysate, a Cell Counting Kit-8 (CCK-8) and DAPI Stain Solution were purchased from Beyotime (Shanghai, China). Anti-CD45 antibody, anti-CD3 antibody and anti-CD8 antibody were purchased from eBioscience (San Diego, California, USA). Anti-HIF-1α antibody was obtained from Abcam (Cambridge, UK). HypoxyprobeTM-1 was obtained from Hypoxyprobe (Massachusetts, USA). Anti-PD-L1 and anti-granzyme B antibodies were purchased from BioLegend (California, USA). Anti-TNF-α and anti-INF-γ antibodies were purchased from Bio-Techne (Minnesota, USA).

**Mouse and cell lines**

Female C57BL/6 mice and BALB/c nude mice were provided by the Qinglongshan Animal Breeding Farm (Nanjing, China) and maintained in a specific pathogen-free environment. MKN45 and MKN28 cells were obtained from the Cell Bank of Chinese Academy of Sciences (Shanghai, China). Cells were added to RPMI-1640 medium containing 10% FBS and 1% penicillin-streptomycin, and cultured in a constant-temperature incubator with a 5% carbon dioxide atmosphere at 37 ℃.

**Characterization of TH-302 NPs**

The average size and zeta potential of TH-302 were measured by the dynamic light scattering method with a nanoparticle tracking analyzer. The morphology and size of mPEG-PLGA NPs and TH-302 NPs were photographed by transmission electron microscopy (TEM; JEM-200CX, JEOL, Tokyo, Japan) and scanning electron microscopy (SEM; JCM-5700, JEOL, Tokyo, Japan). To determine the drug encapsulation efficiency (EE) and loading efficiency (LE) of TH-302 NPs, the drug content in TH-302 NPs was determined by high-performance liquid chromatography. The drug EE and LE were calculated by the following formulas: EE% = (weight of TH-302 in the NPs/weight of feeding TH-302) × 100%; LE% = (weight of TH-302 in the NPs/weight of the NPs) × 100%.

**Cell viability assay**

Cell viability was evaluated by the CCK-8 assay. Cells were inoculated into 96-well plates and allowed to adhere to the wall for 24 h. TH-302 and TH-302 NPs were added at the indicated concentrations (2, 4, 6, 8, 10, 20, 50 and 100 μmol/L), and the cells were incubated under the specified hypoxic or normoxic conditions for 48 h. After adding 10 μL of CCK-8 reagent to each well, we placed the culture plate in the incubator for 2 h and determined the absorbance (OD value) at 450 nm with a microplate reader (BioTek, USA).

**Apoptosis assay**

MKN45 and MKN28 cells were cultured with PBS, TH-302 or TH-302 NPs under normoxic or hypoxic (1% O_2_, 5% CO_2_) conditions for 48 h. Then, the cells were collected for annexin V-FITC/PI double staining and detected by flow cytometry.

**Colony formation assay**

MKN45 and MKN28 cells were inoculated into 6-well plates 24 h before treatment to allow them to fully adhere to the wall. Under hypoxic or normoxic conditions, the cells were incubated with TH-302 and TH-302 NPs for 48 h. After cultured under normoxic conditions for 7~10 d, the cell colonies were fixed and stained with crystal violet. Clones containing more than 50 cells were counted.

**Western blot**

The harvested cells were washed with PBS and then lysed in RIPA buffer. The supernatant was collected after centrifugation, and 5 × SDS loading buffer was then added. The mixture was boiled for 10 min. The proteins were separated by SDS-PAGE and transferred to polyvinylidene fluoride membranes. The membranes were probed with primary antibodies overnight at 4 °C. After incubation with the secondary antibody for 2 h at room temperature, the membranes were washed and visualized using a chemiluminescence machine (Bio-Rad, USA). The band density was quantified using Image Lab software (Bio-Rad, USA).

**Flow cytometry analysis of cell membrane PD-L1**

The cells were centrifuged for 5 min and collected. After incubation with the anti-PD-L1 antibody in the dark for 30 min at 4 °C, the cells were resuspended in FACS washing buffer and subjected to flow cytometry analysis. The data were analyzed with FlowJo software (FlowJo LLC, USA).

**Tumorigenesis experiment in BALB/c nude mice**

A total of 2 × 10^6^ MKN45 cells were resuspended in 100 μL of PBS and then subcutaneously inoculated into the groin of the left lower limb of BALB/c nude mice. Then the tumour-bearing mice were randomly divided into three groups, with three mice in each group. A dose of 50 mg/kg of TH-302 was injected into mice through the tail vein twice a week. Normal saline was injected into mice in the control group. The tumour length (L) and width (W) were routinely measured using a Vernier calliper for all groups every three days during the treatment cycle. The tumour volume (mm^3^) was calculated by the formula: L × W^2^/2. After treatment, the mice were sacrificed and tumour tissues were harvested. The tumour samples were weighed and used for further experiments.

**Flow cytometry analysis**

After the mice were killed, the tumours were extracted and homogenized. The tissue fragments were then incubated in RPMI-1640 medium containing 5 mg/mL collagenase for 60 min. The homogenate was washed with PBS and then passed through a 70 μm nylon mesh filter. Then the cells were centrifuged for 10 min and the supernatant was discarded to obtain a single-cell suspension. For CD8^+^ T lymphocyte and cytokine analysis, cells were stained with fluorescently labelled anti-CD45, anti-CD3, anti-CD8, anti-TNF-α, anti-INF-γ and anti-granzyme B antibodies at room temperature for 30 min and analyzed by flow cytometry. Further analysis of the data was conducted using FlowJo software.

**Supplementary Figures**

**
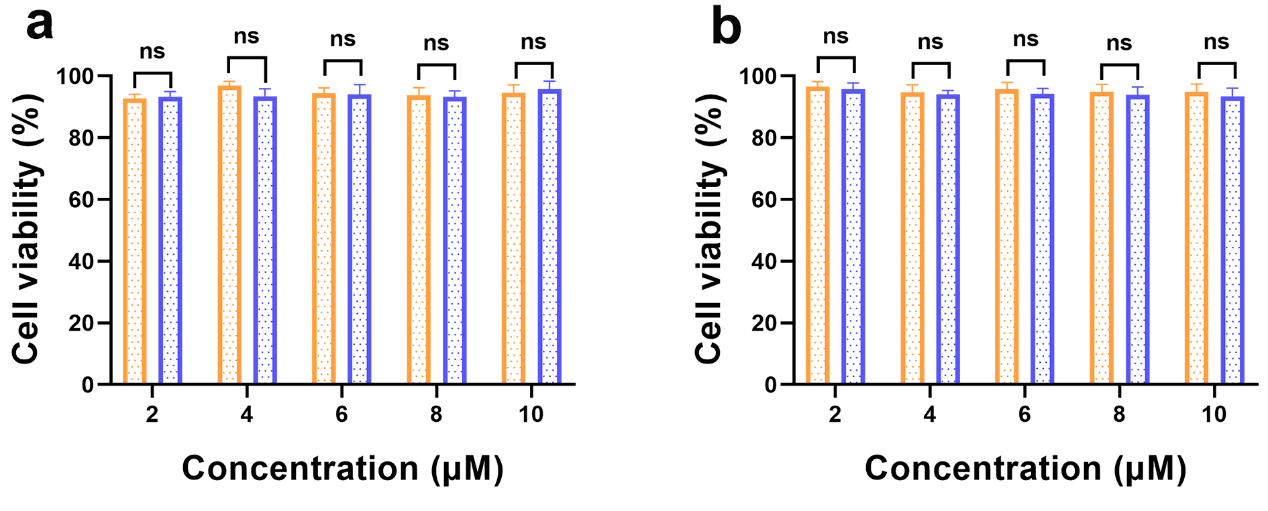
**

**Fig. S1** TH-302 and TH-302 NPs have no cytotoxicity to MKN45 (**a**) and MKN28 (**b**) cells under normoxic conditions.

**
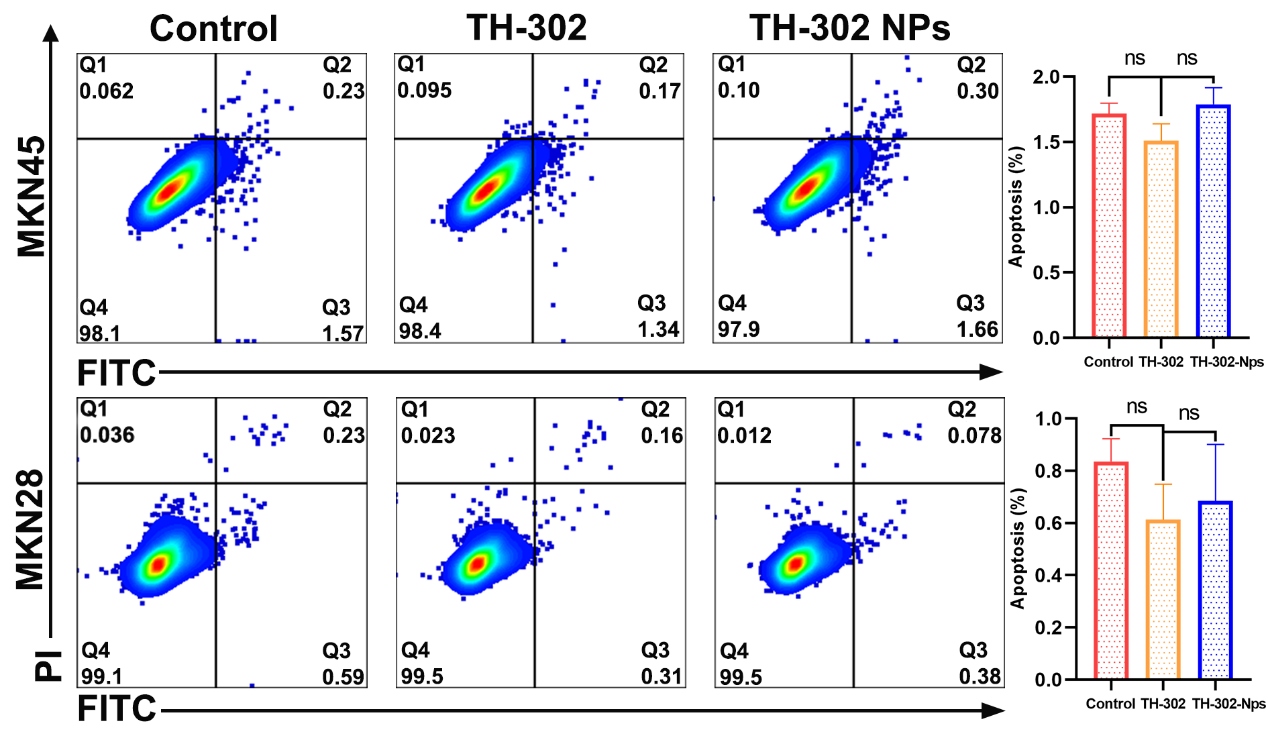
**

**Fig. S2** The Effects of TH-302 and TH-302 NPs on gastric cancer cells apoptosis under normoxic conditions.

**
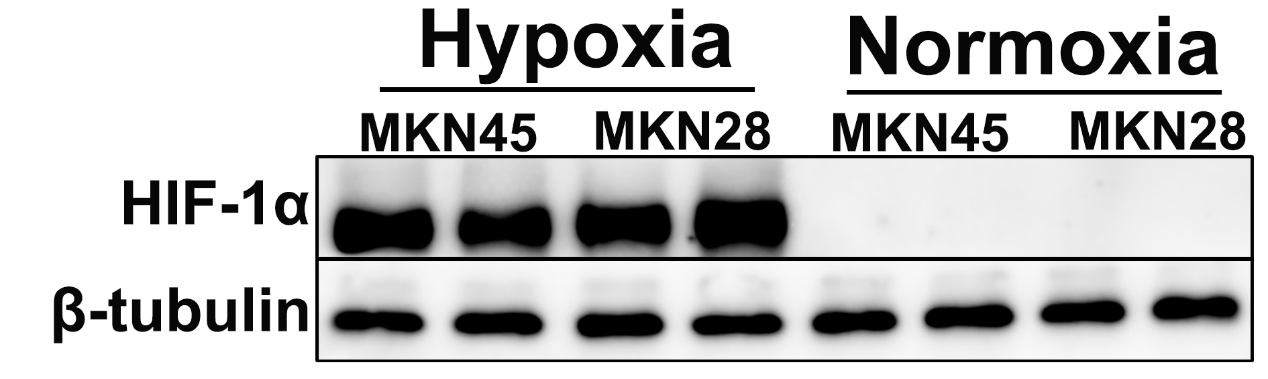
**

**Fig. S3** The expression of HIF-1α in MKN45 and MKN28 cells under hypoxic and normoxic conditions.

**
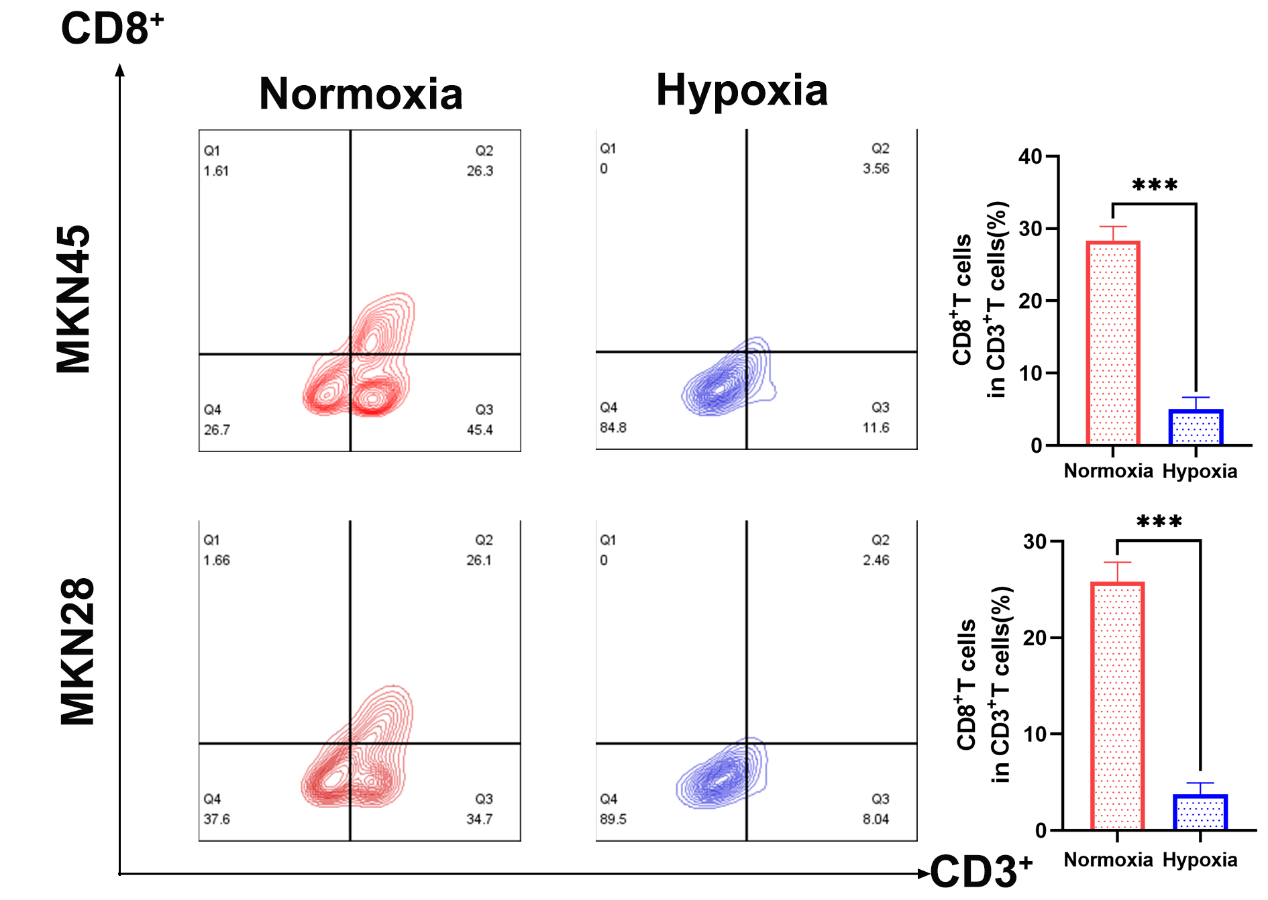
**

**Fig. S4** Hypoxia inhibits the proportion of CD3^+^CD8^+^ cells in PBMCs.

**
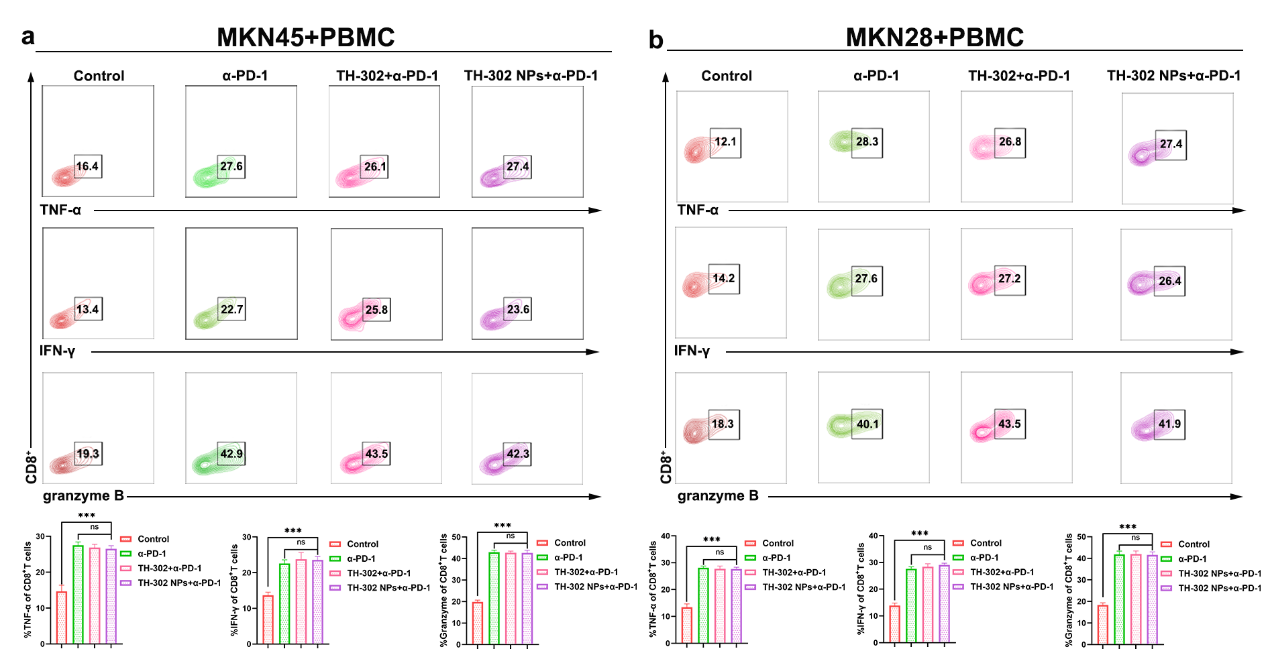
**

**Fig. S5** The effects of TH-302 and TH-302 NPs on cytokine expression secreted by PBMCs cocultured with MKN45 (**a**) and MKN28 (**b**) cells under normoxic conditions.

**
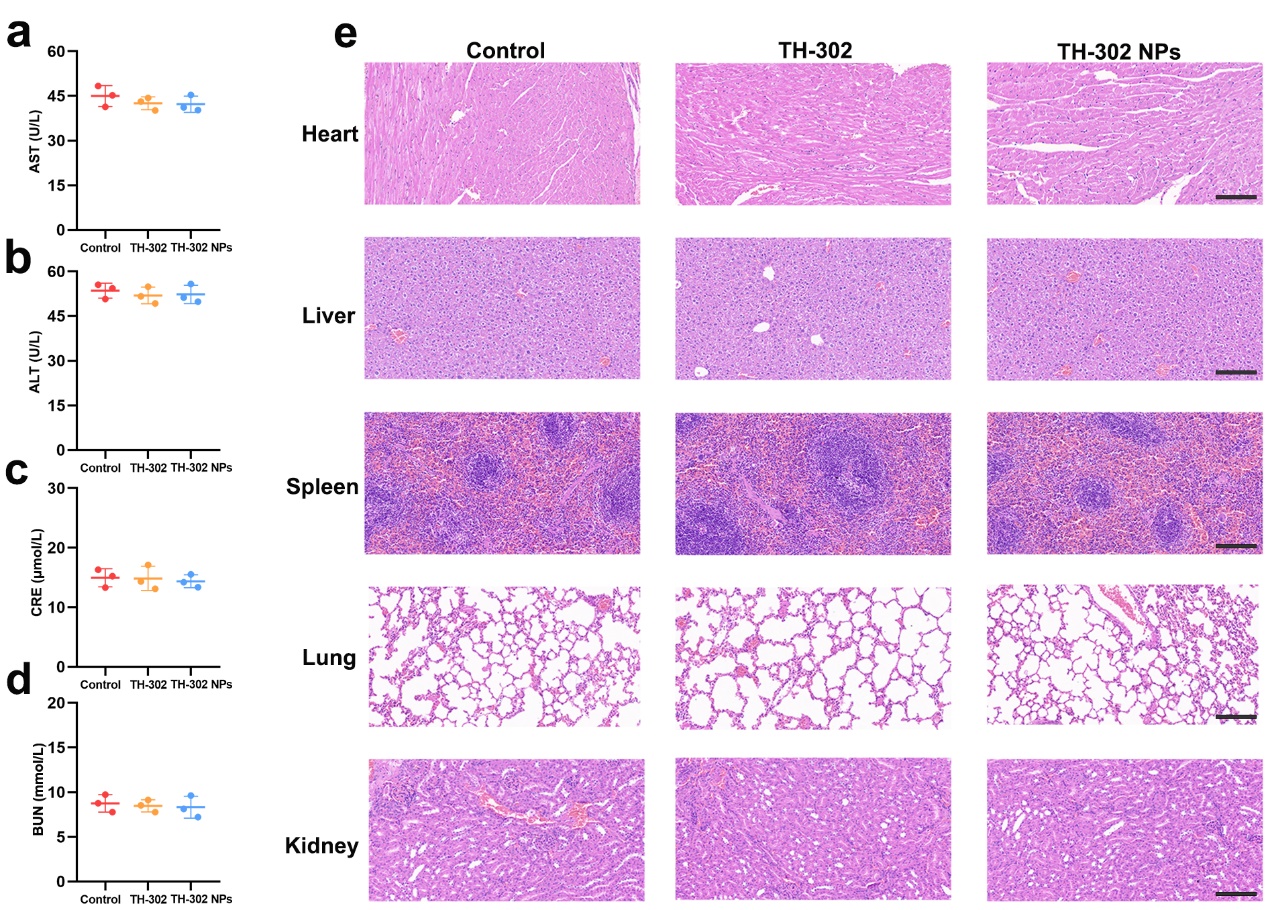
**

**Fig. S6** Biosafety evaluation of TH-302 NPs in BALB/c nude mice. **a-d** The levels of serum AST (a), ALT (b), CRE (c)and BUN (d) in the BALB/c nude mice in the different treatment groups. **e** Representative images of mouse heart, liver, spleen, lung, and kidney sections of BALB/c nude mice stained with H&E staining. Scale bar: 100 μm.
